# Efficacy and Immune-Related Adverse Events of Immune Checkpoint Inhibitors in Patients With Non-small Cell Lung Cancer and Autoimmune Diseases

**DOI:** 10.7759/cureus.85067

**Published:** 2025-05-29

**Authors:** Kenta Takashima, Kazutoshi Isobe, Hiroki Wakabayashi, Kaichi Kaneko, Atsuhito Saiki, Yasuo Matuzawa

**Affiliations:** 1 Department of Internal Medicine, Toho University Graduate School of Medicine, Tokyo, JPN; 2 Department of Internal Medicine, Toho University Sakura Medical Center, Chiba, JPN

**Keywords:** autoimmune disease, autoimmune disease exacerbation, immune-checkpoint inhibitors, immune-related adverse events (iraes), non-small-cell lung cancer

## Abstract

Background: Autoimmune diseases (AIDs) are often associated with lung cancer, but their clinical characteristics remain unclear.

Purpose: The study aimed to elucidate the clinical features of non-small cell lung cancer (NSCLC) in patients with AIDs.

Methods: This single-center, retrospective observational study was conducted between June 2016 and October 2024. It included patients diagnosed with primary NSCLC and comorbid AIDs who received lung cancer treatment.

Results: A total of 32 patients (median age: 68 years; male/female: 14/18) were enrolled. Rheumatoid arthritis was the most common AID (10 cases, 31.3%), followed by Graves’ disease, Hashimoto’s thyroiditis, and ulcerative colitis (four cases each, 12.5%). Other AIDs included systemic lupus erythematosus (two cases, 6.3%) and one case (3.1%) each of Behçet’s disease, dermatomyositis, myasthenia gravis, scleroderma, Schmidt’s syndrome, psoriasis, and sarcoidosis. Immune checkpoint inhibitors (ICIs) were administered to 11 patients (34.3%), with a response rate and disease control rate of 63.6%. Immune-related adverse events (irAEs) occurred in 72.7% (8/11) of patients, including one grade 3 event. Of which, AID exacerbation related to ICI occurred in 45.5% (5/11) of patients. The median progression-free survival was 126 days, and the median overall survival (OS) was 1,594 days. Among patients without driver mutations who received ICI therapy, the median OS was significantly longer compared to those with driver mutations (2,274 vs. 654 days; P < 0.001). Multivariate analysis revealed that good performance status, adenocarcinoma history, and ICI administration were associated with better OS.

Conclusions: ICIs may be effective in selected NSCLC patients with AIDs; however, careful monitoring is essential due to the risk of irAE and AID exacerbation.

## Introduction

Autoimmune diseases (AIDs) are characterized by excessive immune cell activation that causes inflammation in healthy tissues. These conditions include a wide range of disorders, such as connective tissue diseases, endocrine disorders, gastrointestinal conditions, skin diseases, neurological disorders, and respiratory diseases. According to a study based on the Surveillance, Epidemiology, and End Results (SEER) database, the prevalence of AID among 210,509 lung cancer patients aged 65 years and older in the United States was 13.5% [1]. Rheumatoid arthritis (RA) was the most common (5.9%), followed by psoriasis (2.8%), polymyalgia rheumatica (1.8%), Addison’s disease (1.0%), systemic lupus erythematosus (SLE) (0.9%), ulcerative colitis (UC) (0.8%), and giant cell vasculitis (0.8%) [1].

Despite this high prevalence, patients with non-small cell lung cancer (NSCLC) and comorbid AID are often excluded from large-scale clinical trials, making it difficult to determine appropriate standard treatments. Among 198 clinical trials, 68 excluded all patients with AID, 13 excluded those with active AID, and 87 excluded patients with active AID requiring treatment [2]. As a result, only 69.5% of patients with NSCLC and AID received standard treatment, compared to 97.3% of those without AID (P < 0.001) [3].

Immune checkpoint inhibitors (ICIs), including those targeting programmed death-1 (PD-1)/programmed death-ligand 1 (PD-L1) and cytotoxic T-lymphocyte protein 4 (CTLA-4), have been used to improve survival in patients with metastatic melanoma, renal cell carcinoma, head and neck cancer, and NSCLC. ICIs have become the standard treatment for NSCLC without driver mutations, such as epidermal growth factor receptor (EGFR) mutation and anaplastic lymphoma kinase receptor (ALK) rearrangement [4-11]. However, the safety and efficacy of ICIs in patients with NSCLC and AID remain unclear. This uncertainty is largely due to concerns over an increased risk of immune-related adverse events (irAEs) and the potential for exacerbation of pre-existing AID during ICI therapy [10,12-16]. For example, patients with NSCLC and AID are reported to be at higher risk of developing grade 3 to grade 4 irAEs, and approximately half of RA patients receiving ICIs experience disease exacerbation [13]. In this study, we conducted a retrospective analysis to clarify the clinical features of NSCLC in patients with AID.

## Materials and methods

Study design and participants

This single-center, retrospective study was conducted at Toho University Medical Center Sakura Hospital, Sakura, Japan, and included patients from January 2016 to October 2024. Eligible patients met the following criteria: (1) pathologically confirmed NSCLC; (2) comorbid AID before lung cancer diagnosis; and (3) receipt of lung cancer treatment, including ICI monotherapy or cytotoxic chemotherapy, or molecular targeted therapy. The exclusion criteria included (1) patients with confirmed diagnoses of paraneoplastic syndromes, and (2) those who refused to opt out. This study aimed to clarify the clinical characteristics of NSCLC in patients with AID. The study protocol was approved by the Ethics Committee of Toho University Sakura Medical Center (approval number: S24113_S24072). Informed consent was waived owing to the retrospective design; however, information about the study was disclosed on the institutional website, providing patients the opportunity to opt out.

Data collection

Clinical data of each patient were obtained from the Toho University Medical Center Sakura Hospital database. The following information was collected from all study participants: age at diagnosis, sex, smoking history, histological diagnosis, clinical stage, Eastern Cooperative Oncology Group (ECOG) performance status (PS), PD-L1 status, presence of EGFR mutation, ALK rearrangement, and lung cancer treatment details (treatment regimen, treatment start date, irAE grade, last follow-up date, and date of death). Data on AID included the AID diagnosis, presence of interstitial pneumonia (IP) and antibodies, and use of glucocorticoids or immunosuppressive medications. Tumor stage was based on the eighth edition of the American Joint Committee on Cancer classification.

AID diagnosis

Included AIDs were classified into six categories: (1) connective tissue diseases such as RA, SLE, Sjögren’s syndrome, dermatomyositis, scleroderma, mixed connective tissue disease, and vasculitis syndromes (including polyarteritis nodosa, microscopic polyangiitis (MPA), granulomatosis with polyangiitis, Takayasu’s arteritis, and giant cell arteritis), as well as Behçet’s disease; (2) endocrine diseases, such as autoimmune thyroiditis and type 1 diabetes; (3) gastrointestinal diseases, including UC, Crohn’s disease, autoimmune hepatitis, primary biliary cholangitis, and primary sclerosing cholangitis; (4) skin diseases, including psoriasis; (5) neurological disorders, including multiple sclerosis; (6) respiratory diseases, including connective tissue disease-related lung disease and sarcoidosis. Each AID was diagnosed by an appropriate board-certified specialist based on the established classification criteria.

Statistical analysis

Tumor response was evaluated according to the Response Evaluation Criteria in Solid Tumors version 1.1. The best response was defined as the most favorable objective tumor response observed during ICI therapy. The objective response rate (ORR) was defined as the proportion of patients achieving either complete response (CR) or partial response (PR). The disease control rate (DCR) was defined as the proportion of patients achieving CR, PR, or stable disease. Progression-free survival (PFS) was defined as the time from treatment initiation to either disease progression or death from any cause. Overall survival (OS) was defined as the time from treatment initiation to death from any cause. Patients lost to follow-up were censored at the last confirmed survival date.

Statistical analyses were performed using IBM SPSS Statistics for Windows, Version 24 (Released 2017; IBM Corp., Armonk, New York, United States). Survival curves were generated using the Kaplan-Meier method, and statistical comparisons were made using the log-rank test. Multivariate Cox proportional hazards regression analysis was conducted to identify factors associated with prolonged OS. Variables included statistical age, sex (male vs. female), PS (0 vs. ≥1), smoking history, path (adenocarcinoma vs. others), clinical stage (III vs. IV vs. surgical reoccurrence), driver mutation (yes vs. no), Tumor Proportion Score of PD-L1, autoantibodies (yes vs. no), operation (yes vs. no), radiation therapy (yes vs. no), prednisolone (PSL) medication (yes vs. no), and IrAE and/or progression (yes vs. no). Additional variables included the number of metastases, IP (yes vs. no), and ICI therapy (yes vs. no). A P-value less than 0.05 was considered statistically significant.

## Results

Demographic characteristics

**Figure 1:**
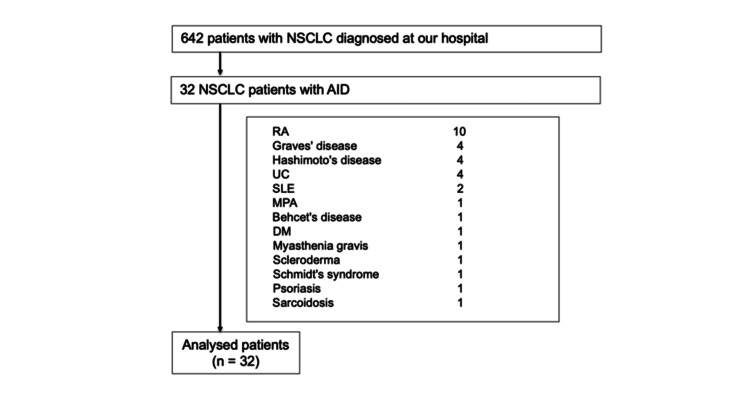
Flow diagram of patient selection and study inclusion criteria NSCLC: non-small cell lung cancer; AID: autoimmune disease; RA: rheumatoid arthritis; UC: ulcerative colitis; SLE: systemic lupus erythematosus; MPA: microscopic polyangiitis; DM: diabetes mellitus

From January 2016 to October 2024, 624 patients diagnosed with lung cancer visited Toho University Medical Center Sakura Hospital. According to the eligibility criteria, 32 patients were included in this study (Figure 1). The mean age of the cohort was 68 years, and 14 patients (43.8%) were male. All patients had an ECOG PS of 0-1, indicating a relatively good functional status at the time of treatment initiation. A history of smoking was observed in 65% of cases, reflecting a typical risk factor for lung cancer. Histologically, the majority of patients (78%) were diagnosed with adenocarcinoma, and seven patients (21.9%) had squamous cell carcinoma. The majority of patients presented with advanced-stage disease (clinical stage III or IV), underscoring the aggressive nature of NSCLC in this cohort. PD-L1 expression ≥50% was observed in only 12.5% of patients, which is relevant for evaluating the potential efficacy of immunotherapy. Driver mutations were detected in 11 patients, with EGFR mutations identified in 10 cases and ALK rearrangement in one case (Table 1).

**Table 1:** Demographic characteristics of the participants (n = 32). Rec: recurrence after surgical resection; PD-L1: programmed death-ligand 1; TPS: Tumor Proportion Score; EGFR: epidermal growth factor receptor; ALK: anaplastic lymphoma kinase receptor

Variables	Values, n (%)
Age (year)	
Median (range)	68 (51-94)
Sex	
Male	14 (43.8)
Female	18 (56.2)
Performance status	
0	17 (53.1)
1	13 (46.9)
Histological findings	
Adenocarcinoma	25 (78.1)
Squamous cell carcinoma	7 (21.9)
Clinical stage	
III	10 (31.2)
IV	17 (53.1)
Rec	5 (15.6)
TNM (stage III, IV)	
T (T4/T3/T2/T1)	4/7/3/13
N (N3/N2/N1/N0)	4/2/6/13
M (T0/M1)	10/17
Smoking history	
Current	5 (15.6)
Former	16 (50.0)
Never	11 (34.4)
PD-L1 expression (TPS%)	
50-100	4 (12.5)
1-49	4 (12.5)
0	5 (15.6)
Unknown	19 (59.4)
EGFR mutation	
Positive	10 (31.2)
Negative	17 (53.1)
Unknown	5 (15.6)
ALK rearrangement	
Positive	1 (3.1)
Negative	8 (25.0)
Unknown	23 (71.9)

AID

The distribution of AID types is summarized in Table 2. RA was the most commonly seen in 10 patients (31.3%). Other AIDs included Graves’ disease (four cases, 12.5%), Hashimoto’s thyroiditis (four cases, 12.5%), UC (four cases, 12.5%), SLE (two cases, 6.3%), and one case (3.1%) each of Behçet’s disease, dermatomyositis, myasthenia gravis, scleroderma, Schmidt’s syndrome, psoriasis, and sarcoidosis. IP was present in five patients (one with idiopathic pulmonary fibrosis (IPF) and four with non-specific interstitial pneumonia (NSIP)). Glucocorticoids were administered in nine cases (28.1%), and immunosuppressants in 11 cases (34.3%). Moreover, ICIs were used in 11 cases (34.3%).

**Table 2:** Autoimmune diseases diagnosed among the participants (n = 32). RA: rheumatoid arthritis; UC: ulcerative colitis; SLE: systemic lupus erythematosus; MPA: microscopic polyangiitis; DM: dermatomyositis; IP: interstitial pneumonia; IPF: idiopathic pulmonary fibrosis; NSIP: non-specific interstitial pneumonia

Autoimmune diseases	n (%)
RA	10 (31.3)
Graves' disease	4 (12.5)
Hashimoto's disease	4 (12.5)
UC	4 (12.5)
SLE	2 (6.3)
MPA	1 (3.1)
Behcet's disease	1 (3.1)
DM	1 (3.1)
Myasthenia gravis	1 (3.1)
Scleroderma	1 (3.1)
Schmidt's syndrome	1 (3.1)
Psoriasis	1 (3.1)
Sarcoidosis	1 (3.1)
IP	
IPF	1 (3.1)
NSIP	4 (12.5)
Non	27 (84.4)
Antibody	
Yes	24 (75.0)
No	8 (25.0)
Glucocorticoids	
Yes	9 (28.1)
No	23 (71.9)
Immunosuppressive drugs	
Yes	11 (34.3)
No	21 (65.7)

IrAE and AID progression with ICI therapy

Table 3 summarizes patients receiving ICI treatments. Seven patients received single-agent ICI therapy, and four received ICIs in combination with cytotoxic agents. These treatments were primarily administered as first- or second-line therapy. Table 4 summarizes irAEs and grades. Overall, any grade of irAEs was observed in 8/11 patients (72.7%), and grades 3-5 irAEs occurred in three patients (27.3%). Among them, AID progression was observed in four patients: UC (two cases), dermatomyositis (one case), and RA (one case). Additional glucocorticoids were introduced in four cases. ICI therapy was discontinued in three of the five patients who experienced either irAEs or AID exacerbation (Table 5).

**Table 3:** Immune checkpoint inhibitor (ICI) therapies among the participants (n = 11). CBDCA: carboplatin; PEM: pemetrexed; PTX: paclitaxel; BEV: bevacizumab

ICI monotherapy	7
Pembrolizumab	2
Nivolumab	3
Atezolizumab	1
Durvalumab	1
ICI (+ICI) + platinum doublet	4
CBDCA + PEM + pembrolizumab	1
CBDCA + PTX + atezolizumab + BEV	2
CBDCA + PEM + ipilimumab + nivolumab	1

**Table 4:** Immune-related adverse event (irAE) among the participants (n = 11).

irAE	All grades	Grade ≥3
Diarrhea	3 (27.3%)	2 (18.2%)
Agranulocytosis	1 (9.1%)	1 (9.1%)
Hyperparathyroidism	1 (9.1%)	0
Myositis	1 (9.1%)	0
Pneumonitis	1 (9.1%)	0
Arthritis	1 (9.1%)	0

**Table 5:** Autoimmune disease (AID) progression related to ICI therapies among participants (n = 5). ICI: immune checkpoint inhibitor; DM: dermatomyositis; UC: ulcerative colitis; RA: rheumatoid arthritis; PSL: prednisolone

AID	Additional medication	ICI therapy
DM	PSL 20 mg/day	Continue
UC	Non	Discontinue
UC	PSL 10 mg/day	Discontinue
UC	PSL 30 mg/day	Discontinue
RA	PSL 20 mg/day	Continue

Efficacy of ICI therapy

**Figure 2:**
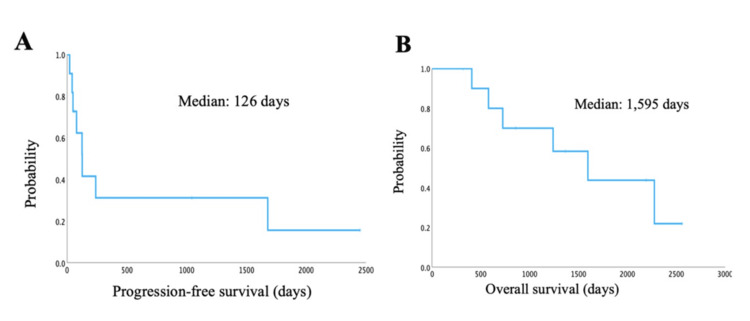
Survival outcomes with immune checkpoint inhibitor (ICI) therapy. (A) Progression-free survival (PFS); (B) Overall survival (OS).

**Figure 3:**
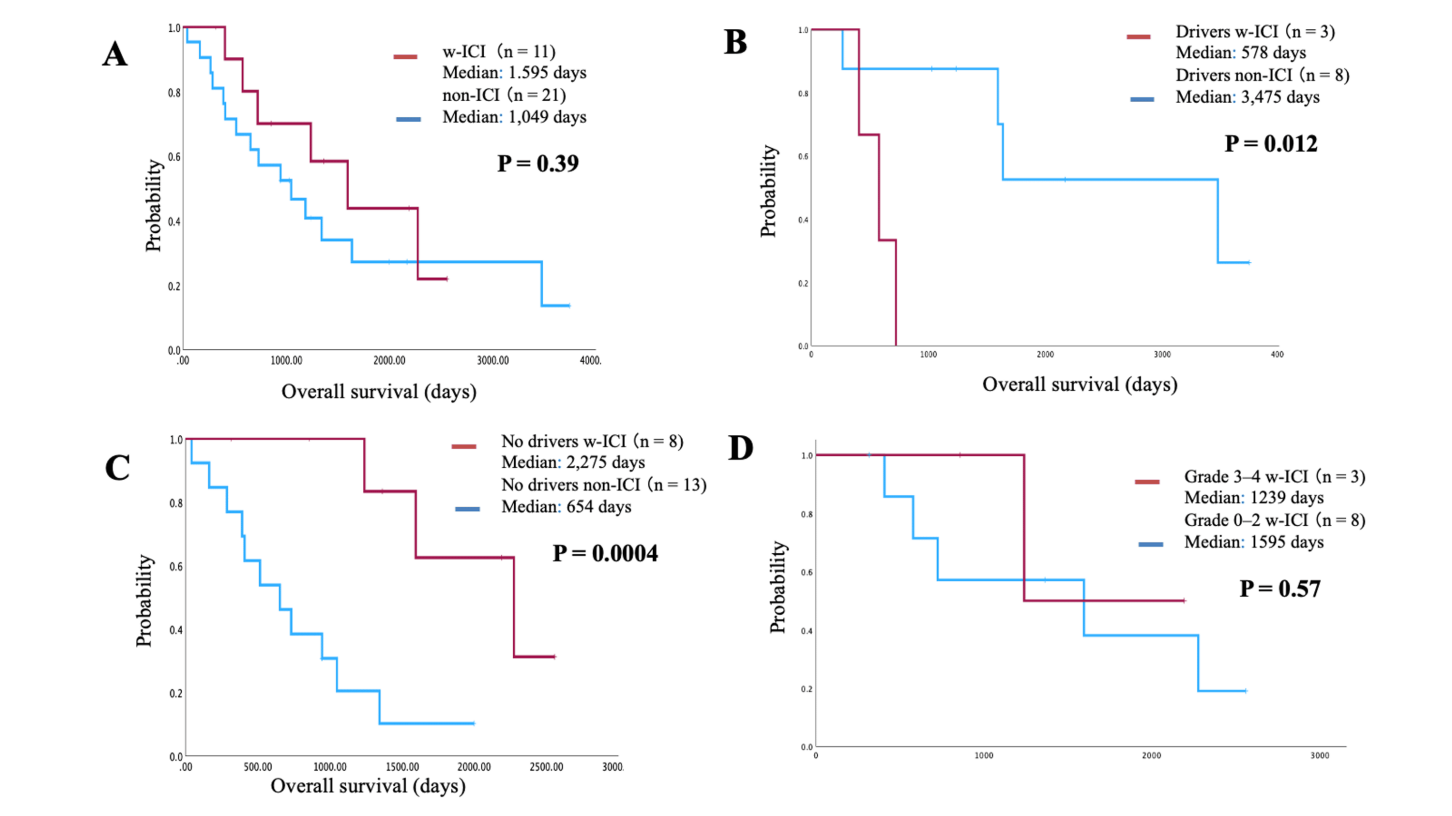
Efficacy of immune checkpoint inhibitor (ICI) therapy in patients with and without driver mutations. (A) No significant difference in overall survival (OS) was observed between ICI- and non-ICI-treated patients (median OS: 1,595 vs. 1,049 days, P = 0.39). (B) A significant difference in OS was observed between non-ICI and ICI therapy in patients with driver mutations (578 days with ICI vs. 3,475 days without ICI, P = 0.012). (C) However, in patients without driver mutations, OS was significantly longer in the ICI-treated group than in the non-ICI group (2,275 days with ICI vs. 654 days without ICI, P = 0.004). (D) There was no significant difference in OS between patients with grade ≥3 immune-related adverse events (irAEs) and those with grades 0-2 irAEs (grade ≥3 OS: 1,239 days with ICI vs. grades 0-2 OS: 1,595 days without ICI, P = 0.57).

Among the 11 patients who received ICI therapy, CR was achieved in four cases, PR in three cases, and progressive disease (PD) in four cases. The ORR and DCR were both 63.6%. The median PFS was 126 days, and the median OS after ICI treatment was 1,595 days (Figures 2A, 2B). No significant difference in OS was observed between ICI-treated and non-ICI-treated patients (median OS: 1,595 vs. 1,049 days, P = 0.39) (Figure 3A). However, a significant difference was found for OS between non-ICI therapy and ICI therapy in patients with driver mutation (578 days with ICI vs. 3,475 days without ICI, P = 0.012) (Figure 3B). In patients without driver mutations, OS was significantly longer in the ICI-treated group than the non-ICI group (2,275 days with ICI vs. 654 days without ICI, P = 0.004) (Figure 3C). There was no significant difference in OS between patients with grade ≥3 irAEs and those with grades 0-2 irAEs (grade ≥3 OS: 1,239 days with ICI vs. grades 0-2 OS: 15,954 days without ICI, P = 0.57) (Figure 3D).

Prognosis based on the presence of autoantibodies

**Figure 4:**
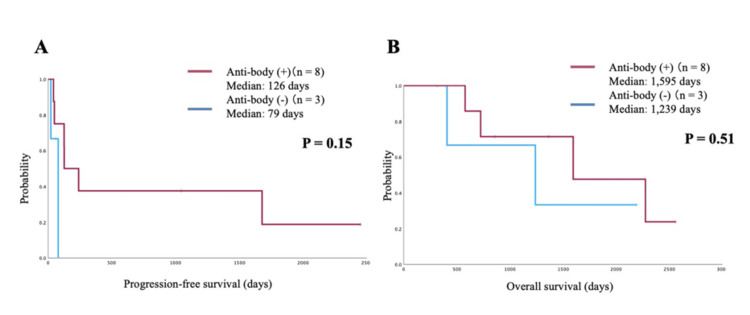
Prognosis based on the presence or absence of autoantibodies. (A) Progression-free survival (PFS) based on autoantibody status. (B) Overall survival (OS) based on autoantibody status.

Autoantibodies were detected in 8/11 patients who received ICI therapy. No significant difference in prognosis was observed between patients with and without autoantibodies (PFS: 126 days vs. 79 days, P = 0.15; OS: 1,595 days vs. 1,239 days, P = 0.51) (Figures 4A, 4B).

Indicators of longer OS

Multivariate Cox regression analysis identified PS (hazard ratio (HR): 6.610; 95% confidence interval (CI): 1.893-23.075; P = 0.003), number of metastases (HR: 1.675; 95% CI: 1.175-2.387; P = 0.004) and receipt of ICI therapy (HR: 4.912; 95% CI: 1.116-21.621; P = 0.004) as significant indicators of longer OS (Table 6).

**Table 6:** Indicators for longer overall survival (n = 32). PS: performance status; irAE: immune-related adverse event; Path: pathological type; Ad: adenocarcinoma; Sq: squamous cell carcinoma; PSL: prednisolone; IP: interstitial pneumonia; ICI: immune checkpoint inhibitor

Indicator	Hazard ratio		95% CI	P-value
PS (0 vs. 1 vs. 2)		6.610	1.893-23.075	0.003
Path (Ad vs. Sq)		0.116	0.03-0.450	0.02
PSL medication (yes vs. no)		1.861	0.560-6.186	0.311
IrAE and/or progression (yes vs. no)		0.030	0.002-0.404	0.008
Number of metastases		1.675	1.175-2.387	0.004
IP (yes vs. no)		1.323	1.175-2.387	0.171
ICI therapy (yes vs. no)		4.912	1.116-21.621	0.035

## Discussion

In this study, patients without driver mutations who received ICI therapy had a significantly better prognosis than those who did not receive ICI therapy (median OS: 2,275 days vs. 654 days, P < 0.001). Multivariate analysis further confirmed that ICI therapy was independently associated with improved OS.

Previous studies have reported that the incidence of lung cancer in patients with AID is approximately 13.5% [1]. In our cohort, the incidence was 5.1% (32/624 cases), suggesting a relatively lower rate of AID among lung cancer patients at our center. This discrepancy may be due to our inclusion criteria, which focused on patients who had received treatment for lung cancer.

Additionally, the distribution of AID types in our study revealed a lower incidence of RA, a higher incidence of UC, and a similar rate of SLE compared to previous reports. These variations are likely influenced by racial or regional differences in disease prevalence.

The first issue to highlight is that ICI treatment has traditionally been avoided in lung cancer patients with concomitant AID. In this study, ICIs were administered as monotherapy in seven patients and in combination with cytotoxic chemotherapy in four patients. Overall, ICI treatment was used in 38% of patients (8/21 cases), which is notably lower than the 69.5% reported in previous studies [3].

The second concern involves the safety of ICI therapy in this patient population. Previous reports have indicated that the incidence of irAEs in lung cancer patients with AID is 45.1% for all grades and 23.9% for grade ≥3 irAEs [14]. In this study, irAEs of any grade occurred in 72.4% (8/11 cases), and grade ≥3 irAEs were observed in only 27.3% (3/11 cases), suggesting a relatively higher incidence of irAEs in our cohort.

The third issue is the potential exacerbation of pre-existing AID during ICI treatment. Previous reports have reported AID flare-ups in 25.4% to 42.0% of patients receiving ICIs [13,17]. In our study, AID progression occurred in 45.4% (5/11 cases). Most patients improved with the addition of glucocorticoids, but ICI therapy was discontinued in 60% (3/5 cases) due to AID exacerbation. Other studies have reported that AID exacerbations can often be managed with glucocorticoids, but caution is needed. Approximately 21% of patients in these studies permanently discontinue ICI treatment, with some requiring immunosuppressants or experiencing fatal outcomes [13,17]. Despite these risks, numerous reports support that ICIs can generally be administered safely in lung cancer patients with AID under careful monitoring [16,18].

Notably, ICI treatment appears to be effective in patients with lung cancer and coexisting AID. In this study, ICI therapy emerged as a significant independent prognostic factor for prolonged OS (HR: 4.912; 95% CI: 1.116-21.621; P = 0.004). Supporting this finding, the NEJ 047 study also reported that immune checkpoint blockade extended survival in patients with lung cancer and AID (HR: 0.43; 95% CI: 0.26-0.70; P = 0.0006) [14]. These findings suggest a potential survival benefit of ICI therapy in this patient population. However, prospective clinical trials are necessary to confirm the efficacy and safety of ICI treatment in lung cancer patients with AID.

Several studies have reported that autoantibodies in NSCLC patients are associated with the efficacy of PD-1 inhibitors [19-21]. Toi et al. demonstrated that patients with pre-existing autoantibodies had significantly longer PFS compared to those without, and that irAEs were more frequent among these patients. Their multivariate analysis indicated that the presence of pre-existing autoantibodies was independently associated with the development of irAEs (OR: 3.25; 95% CI: 1.59-6.65; P = 0.001) [20]. Additionally, other studies have shown that the presence of such antibodies is significantly associated with the occurrence of grade ≥2 irAEs and that patients with autoantibodies experience earlier and more frequent irAEs when treated with ICIs [21].

In this study, no significant difference in prognosis was observed based on the presence or absence of autoantibodies (PFS: 126 vs. 79 days, P = 0.15; OS: 1,595 vs. 1,239 days, P = 0.51; data not shown). These findings suggest the need for further investigation into the relationship between specific autoantibody types, ICI efficacy, and the incidence of irAEs.

This study has several limitations. It was a single-center, retrospective study with a small sample size, and the diagnosis of AID was based on clinical judgment. As this is a single-center study, the findings may have limited generalizability. Additionally, patients with AID who received treatment at other hospitals may not have been captured in our database; thus, they were excluded from the study. Although certain AIDs, such as myositis and myasthenia gravis, have been reported as paraneoplastic syndromes [22], distinguishing between the two was challenging in this study. Larger prospective studies are needed to confirm the effectiveness and safety of ICI in patients with NSCLC and AID.

## Conclusions

In conclusion, this study highlights the potential benefit of ICI therapy in NSCLC patients with AID, particularly in those without driver gene mutations. Although OS was similar between ICI- and non-ICI-treated patients when considering all patients, a significant survival benefit of ICI therapy was observed in those without driver mutations. These findings suggest that ICI treatment can offer substantial survival benefits, particularly in this subgroup, despite the complexities involved in managing AID and irAEs. AID progression during ICI treatment, although concerning, was manageable in most cases with glucocorticoid therapy. However, caution is necessary to prevent permanent discontinuation of therapy.

These results underscore the importance of carefully considering the use of ICIs in lung cancer patients with coexisting AID, and emphasizing the need for individualized risk management strategies. Future prospective studies are needed to further assess the long-term safety and efficacy of ICI therapy in this patient population. Additionally, investigating the relationship between pre-existing autoantibodies and ICI efficacy, along with understanding the mechanisms behind irAEs in AID patients, will be crucial for optimizing treatment strategies and improving patient outcomes in this complex clinical setting.
